# The Effect of Mother’s Voice on Arterial Blood Sampling Induced Pain in Neonates Hospitalized in Neonate Intensive Care Unit

**DOI:** 10.5539/gjhs.v7n6p198

**Published:** 2015-04-16

**Authors:** Elham Azarmnejad, Forogh Sarhangi, Mahrooz Javadi, Nahid Rejeh

**Affiliations:** 1 Faculty of Nursing, Baqiyatallah university of Medical Sciences, Tehran, Iran; 2 Pediatric Nursing, Faculty of Nursing, Baqiyatallah University of Medical Sciences, Tehran, Iran; 3 Elderly Care Research Center, Department of Nursing, Faculty of Nursing and Midwifery, Shahed University, Iran

**Keywords:** neonate, pain, mother’s voice

## Abstract

**Background and Objective:**

Due to devastating effects of pain in neonates, it is very important to ease it though safe and feasible methods. This study was to determine the effect of familiar auditory stimuli on the arterial blood sampling (ABS) induced pain in term neonates.

**Research Method:**

This study was done on 30 newborns hospitalized in neonate intensive care unit (NICU) of a hospital in Tehran. Research samples were selected by using convenience sampling and randomly divided into two groups of control and test. In the test group, the recorded mothers’ voices were played for the newborns before and after blood sampling procedure. Then, pain measures were recorded 10 minutes before, during and 10 minutes after blood collection based on Neonatal Infant Pain Scale (NIPS); then the pain level changes were reviewed and studied.

**Findings:**

The findings showed significant differences between the control and test groups that indicating the effect of mother’s voice on reducing the pain of neonates during the ABS (p<0.005).

**Conclusion:**

Research findings demonstrate that mother’s voice reduces ABS induced pain in the term neonates.

## 1. Introduction

At birth, critically ill neonates frequently undergo numerous painful procedures as a part of Diagnostic and therapeutic methods for health (Slater, Fabrizi, Worley, Meek, Boyd & Fitzgerald, 2010). Neonates are exposed to approximately 16 invasive procedures per day in NICU during the first two weeks after birth; that only a third of them are done under analgesia (Carbajal et al., 2008).

Until the 1970s, it was believed that neonates are unable to perceive the pain due to incomplete sensory neurons and undeveloped pain receptors. Therefore, painful procedures were applied to newborns without administration of anesthetic and analgesic (Abbasi & Rashidi, 2013). Research has shown that newborn infants can feel pain and remember it. In addition, neonates are more sensitive than adults to pain due to the lack of descending control system, which has a role in easing the pain (Azari, Dargahi, & Mardi, 2011), and they show it through behavioral, physiological, and neural-chemical forms (Mollahadi, Sarhangi, Ebadi & Tadrisi, 2011).

Pain control of neonates is very essential; Due to the high activity of sensory region of newborns’ brain. In addition, full development of pain transmission pathway in newborns, and inappropriate development of pain inhibitory systems cause adverse pain experiences remain in the neonate’s mind (Ta’avoni, Shahali, Haghani & Neisani, 2008).

Appropriate pain management requires huge amount of investigation into and measurement of pain, performing of which accurately and reliably is very difficult, especially with respect to newborns (Parry, 2011). The first step for right diagnosis and effective treatment in newborns is a valid and reliable tool to assess pain. Two of the main limitations to relieve pain at the bedside, are the lack of a pain reliable biomarker and absence of a standard scale that be able to measure the pain intensity (Raeside, 2011).

Newborns’ responses to pain may dramatically differ; therefore, pain assessment tools can be considered as an aid in pain evaluation and management (Parry, 2011). Over the last three decades, many pain scoring tools were defined on the assumption that pain cannot be ignored in newborn infants (Holsti, Grunau, Oberlander & Osiovich, 2008). An ideal pain scoring tool should be widely applicable and convenience, and produce replicable and non-invasive results (Parry, 2011).

Neonates use a variety of behavioral responses to communicate with their caregivers, but Sometimes the actual definition of this responses is subjective that reduce the sensitivity and specificity of these parameters as pain indicators (Elias, Guinsburg, Peres, Balda & Santos, 2008). Crying, as well as body and facial expressions are the main behavioral responses to pain in neonates. The facial expressions reflect specific and effective painful experiences (Grunau, Johnston, & Craig, 1990). In fact, facial expressions have shown the best correlation with cortical activity during a painful stimulus in comparison with physiologic indicators (Slater, Cantarella, Franck, Meek & Fitzgerald, 2008). Facial expressions of Pain include: lowered brows drawn together, eye squeeze, deep forehead creases, lip opening, mouth stretching, tongue tightening, lips pursing and voice trembling (Elias, Guinsburg, Peres, Balda & Santos, 2008).

NIPS is a behavioral scale for pain assessment in newborns, developed and used by Lawrence and colleague in 1993 (Hanson et al., 2010). This scale can measure pain in pre-term and term neonates until six weeks after the birth (Williams, Khattak, Garza & Lasky, 2009). It investigates crying, facial expressions, respiratory pattern, movements of hands and legs, and Level of consciousness in newborn infants (Mollahadi et al., 2011). Reliability and validity of this scale has been confirmed by Suraseranivongse and Colleagues in a study about comparing validity and reliability of three pain scales in neonates (NIPS, CRIES, and CHIPPS) (Suraseranivongse et al., 2006). The validity and reliability of its Persian version has also been confirmed in Iran by Mollahadi (Mollahadi et al., 2011).

The number of interventions is very high in the neonates hospitalized in NICU. There are advanced and effective methods for controlling the pain caused by surgery and major procedures in neonates; while, there is not any appropriate pain control method for minor procedures. Therefore, appropriate pain reduction techniques should be employed during painful procedures (Saki, Mohsenzadeh, Tarrahi & Saki, 2008).

Pain can effectively be managed and reduced by limiting the exposure to the painful stimuli, through both pharmacological and non-pharmacological techniques (Riddell et al., 2011). Thus, due to the harmful complications of the central pain relief medications, pharmacotherapy is less used (Abbasi & Rashidi, 2013).

None-medicinal approaches include a string of innovative activities, done by the patient or caregiver, which safely reduces the patient’s pain or makes it bearable. Today, non-medicinal pain relief methods have attracted nursing systems as well as patients. In addition, these kinds of interventions are effective, simple and safe, and do not depend on specific time or costly equipment. Also, non-medicinal pain relief techniques are free of medicinal complications (Sadeghi, Mohammadi, & Daremi, 2012). Pain relief techniques based on mother-neonate interaction reduce neonatal pain and stress (Ta’avoni & Shahali, 2008).

Auditory responses evolve in the auditory cortex and brainstem at 26-28th weeks of fetal age (Eskandari, Keshavarz, & Jahdi, 2010). Hearing is one of the first senses that a fetus develops, such that it becomes capable of recognizing and remembering the mother’s voice after 24-33th weeks (Djordjevic, 2010). Fetus memorizes musical characteristics of the mothers’ voice, like tone, by listening to it (Arabin, 2002).

Capability of receiving auditory stimulation in the womb and memorizing it by fetus has permanent impact on brain development and self-adjusting in the future (Campbell-Yeo & Marsha, 2011). Any voice that stimulates auditory system is called auditory stimulation (Wilson, 2010).

Mother’s voice is the first and most important low frequency sound audible to the fetus ear (Gooding, 2010). Neonates, at just 3 days of age, can recognize mothers’ voice and heart beats, which affect positively on their physiologic and behavioral responses (Campbell-Yeo & Marsha, 2011). Several studies have investigated the effect of auditory stimuli, such as music or song, on premature neonates. The Results showed a reduction in stress and unstable vital signs, physiologic performance improvement and neural-behavioral development in neonate (Eskandari, Keshavarz, & Jahdi, 2010). The neonate exposed to his/her own mother’s voice has lower heart rate, higher sucking rate, more relaxed look, and less crying and bodily motions (Campbell-Yeo & Fernandes, 2011).

There are few number of studies on the effect of mother’ voice on the severity of ABS induced pain in neonates. Therefore, this study was to determine probable positive effect of this technique, and then recommending it as an operative, cheap and appropriate method to be used in hospitals.

## 2. Research Method

The goal of this study was to determine the effect of mother’s voice (familiar auditory stimuli) on the severity of ABS induced pain in neonates hospitalized in NICU. Inclusion criteria were pregnancy of at least 36 weeks, lack of hearing impairment, not-taking analgesics at least 3 hours prior to sampling, no history of surgery and lack of congenital anomalies and systemic disease. Exclusion criteria were the use of ventilators for neonate and blood sample collection failure.

In this study, a digital scale (vurf model) has been used for weighting the neonate. In addition, NIPS scale was used for measuring pain intensity and Cool Edit 2000 software for recording and playing the sounds. Demographical information of the neonate including age, gender, medical diagnosis, hospitalization duration, type of delivery, APGAR score in 1 and 5 minutes after delivery, weight, and duration of pregnancy (based on week) were recorded in the information form by the researcher. Then, the neonate’s hearing capability was confirmed in response to auditory stimuli by using hearing screening device through OAE^1^ testing. Basic nursing care, changing diapers and feeding the baby were done before the intervention. Then, mother’s voice was recorded and played for the neonate by two small loudspeakers (200V Sony) on both sides of the head at a distance of 20 cm from the neonate’s ear while he/she was in supine position on the Baby Cot in a separate room. The mother’s voice was set at 50-60 dB by using a sound level meter, under the supervision of an audiologist. Then it was played for the intervention group 10 minutes before to 10 minutes after the intervention, in morning shift. The severity of pain was recorded in three stage, 10 minutes before the intervention (before playing the mother’s voice), during the intervention and 10 minutes after the intervention (immediately after completion of the sound). All stages and measures, except sound therapy, were administered to the control group too.

Parents’ participation was voluntarily and the lack of it had no impact on the neonates’ provision with service and care. They were ensured their information is confidential, and also were given consent form.

The data was analyzed by using SPSS 19. Chi-square test was employed to investigate demographic variables qualitatively.

## 3. Findings

In the study was proved the groups were homogeneous by using parametric and non-parametric tests in terms of gender, height, weight, delivery type, pregnancy age, and APGAR score in the 1st and 5th minutes, hospitalization duration and intensity of pain. Among the subjects, 36.66% and 63.33% were respectively, girl and boy. In addition, 16.66% and 83.33% of deliveries were through vaginal delivery and Cesarean section, respectively.

For evaluating the effect of sound therapy on the reduction of neonate pain intensity, was used Generalized Estimating Equation (GEE), with adjusting the values of pain intensity in the zero time.

Based on GEE, the effect of time on pain was not significant (p=0.2). In addition, time-group mutual effect was not significant (p=0.053). In contrast, there was a significant statistical difference between the two groups in terms of pain. In this three stage, pain was significantly lower in the intervention group than control group (p<0.001).

Pain estimation regression score was 3.708 in the intervention group, indicating high probability of pain. This score was 3.708 showing 30 times higher probability of pain in the control group (likelihood of pain was 30 times lower in the intervention group).

**Table 1:** Quantitative data on demographic information of the neonates participated in the study

Demographic information	Mean
Fetal age (week)	2.49 ± 40.4
Height (cm)	3.61 ± 47.38
Weight (g)	495.56 ± 3162.67
1st-minute APGAR score	66 ± 8.88
5th-minute APGAR score	18 ± 9.97
Age at the entry to the study (week)	33.33± 1.06
Hospitalization period (day)	38.33 ± 1.36

**Table 2:** Severity of pain at the baseline (beginning of the intervention), based on NIPS

Statistical test (test statistic) Significance Level	Percentage	Replication number	Pain severity		Group
**Mann-Whitney**	70	21	Sc0	Scores	Control
**U= 0.432**	26.7	8	1		
**z= -0.33**	3.3	1	2		
**P= 0.741**	100	30	Total		
	70	21	0	Scores	Intervention
	13.3	4	1		
	16.7	5	2		
	100	30	Total		

The two groups were equal in terms of initial pain severity.

**Table 3:** Pain severity frequency based on NIPS at the beginning of sound therapy

Percentage	Replication number		NIPS	Group
**3.3**	1	0	Scores	Control
**6.7**	2	3		
**6.7**	2	4		
**33.3**	10	5		
**50**	15	6		
**100**	30	Total		

**6.7**	2	0	Scores	Intervention
**6.7**	2	1		
**3.3**	1	2		
**20**	6	3		
**13.3**	4	4		
**30**	9	5		
**20**	6	6		
**100**	30	Total		

The highest NIPS score was 6, in the control group (50%) and in the intervention group (20%).

**Table 4:** Pain severity based on NIPS, 10 minutes after the completion of sound therapy

Percentage	Replication number	NIPS		Group
**10**	3	3	Scores	Control
**26.7**	8	4		
**16.7**	5	5		
**46.7**	14	6		
**100**	30	Total		

**26.7**	8	0	Scores	Intervention
**13.3**	4	1		
**3.3**	1	2		
**13.3**	4	3		
**13.3**	4	4		
**10**	3	5		
**20**	6	6		
**100**	30	Total		

The highest NIPS scores was 6 in the control group (46.7%) and 0 in the intervention group (26.6%).

**Table 5:** Effect of Sound therapy in reducing the severity of neonate’ pain

Probability (B)	Test hypothesis		Standard Deviation	Overall effectiveness	Parameter

	Significance	Degree of Freedom			
29.715	0.000	1	0.3231	3.708	The effect of intervention factor

## 4. Discussion and Conclusion

The present study showed the effect of mother’s voice on reducing the pain of neonates during arterial blood sampling. So far, no studies have been found about the effect of mother’s voice on the pain level in full term neonates during blood sampling. Johnston, Filion, and Nuyt (2007) assessed neonate born between 32-36 weeks, during routine painful procedures (Heel blood sampling) in NICU. Their study showed no differences in comparing pain responses (PIPP scale) between the two groups. The authors have noted that the mother’s voice recordings level (70 dB) has been higher than predefined levels, and may disturb the neonates. A study was conducted by Butt and Kisilevsky (2000) on comparing the effect of music on premature neonates. The results showed neonates above 31 weeks of age who were exposed to music showed quicker return to their pre-intervention state in terms of facial expressions, used as pain indicators. A study was done by Flippa (2003), on premature neonates that demonstrated live mother’s voice has significant increase in the mean level of saturated arterial oxygen and significant decrease in heart rate in the intervention group comparing control group. Choi et al. (2004) in a study showed that there was significant difference in physiological responses (heart rate, respiratory rate, and oxygen saturation) between the two groups. The intervention group had calmer sleeping behavior. Picciolini and colleague (2014) in a study on newborn infants showed lower heart rate in the intervention group than the control group. Also a study was conducted by Doheny et al. (2012) entitled the effect of mother’s voice on newborn infants hospitalized in NICU that showed shorter improvement period in their physical stability. No study has been done in Iran on the effect of mother’s voice on the pain intensity in premature or full term neonates. Only some studies have been found on the effect of different voices on physiological variables. A study was conducted by Karimi and colleague (2012) entitled “Effect of Music Therapy on Physiological Pain Responses of Blood Sampling in Premature Infants “. they compared the control and test groups in terms of heart rate at the time of needle withdrawal and during the first 5 minutes after it; the results showed significant statistical differences between them (p=0.022 and p<0.005, respectively). A study was done by Eskandari and colleague (2010), entitled “The factors affecting premature neonates’ physiological responses to Quran recitation”. They found increased oxygen saturation, and decreased respiratory and heart rates (p<0.0001) during intervention and 10 minutes after it, that could be indicating of reduced pain level in neonate.

According to these findings, it can be concluded that mother’s voice decreases blood sampling pain in term neonates. Reduction of pain score is the indicator of pain relief between the test and control groups. Therefore, it is recommended to be used in routine painful procedures for which there is not any appropriate medicinal method such as blood sampling, intramuscular injection, venipuncture and so forth. This method is simple, convenience, cheap and most importantly complication-free and practicable technique for easing the pain in neonates.
